# Multiphoton photochemical crosslinking-based fabrication of protein micropatterns with controllable mechanical properties for single cell traction force measurements

**DOI:** 10.1038/srep20063

**Published:** 2016-01-28

**Authors:** Ming Hui Tong, Nan Huang, Wei Zhang, Zhuo Long Zhou, Alfonso Hing Wan Ngan, Yanan Du, Barbara Pui Chan

**Affiliations:** 1 Tissue Engineering Laboratory, Department of Mechanical Engineering, The University of Hong Kong, Pokfulam Road, Hong Kong Special Administrative Region, China; 2 Department of Mechanical Engineering, The University of Hong Kong, Pokfulam Road, Hong Kong Special Administrative Region, China; 3 Department of Biomedical Engineering, School of Medicine, Tsinghua University, Beijing, 100084, China

## Abstract

Engineering 3D microstructures with predetermined properties is critical for stem cell niche studies. We have developed a multiphoton femtosecond laser-based 3D printing platform, which generates complex protein microstructures in minutes. Here, we used the platform to test a series of fabrication and reagent parameters in precisely controlling the mechanical properties of protein micropillars. Atomic force microscopy was utilized to measure the reduced elastic modulus of the micropillars, and transmission electron microscopy was used to visualize the porosity of the structures. The reduced elastic modulus of the micropillars associated positively and linearly with the scanning power. On the other hand, the porosity and pore size of the micropillars associated inversely and linearly with the scanning power and reagent concentrations. While keeping the elastic modulus constant, the stiffness of the micropillars was controlled by varying their height. Subsequently, the single cell traction forces of rabbit chondrocytes, human dermal fibroblasts, human mesenchymal stem cells, and bovine nucleus pulposus cells (bNPCs) were successfully measured by culturing the cells on micropillar arrays of different stiffness. Our results showed that the traction forces of all groups showed positive relationship with stiffness, and that the chondrocytes and bNPCs generated the highest and lowest traction forces, respectively.

The cell niche is known to consist of a complex microenvironment including biological, cellular, and mechanical signals, and it not only serves a structural function, but also stimulates a variety of biomedical and mechanical communications that are necessary for cell attachment, proliferation and differentiation^1^. In recent years, a variety of advanced fabrication technologies have made it possible to precisely engineer the complexities of the cell niche^2^,^3^,^4^. Ink-based writing^5^, for example, uses ‘inks’ prepared from concentrated polyelectrolyte complexes that easily flow through fine nozzles but then rapidly solidify in a coagulation reservoir to achieve defined three-dimensional (3D) patterns. However, with this method, structures are created layer by layer, so their fabrication time may be slow, and they are relatively large, the smallest being several micrometers in size. Holographic lithography^6^, on the other hand, is a rapid and scalable fabrication process for generating 3D periodic microstructures by interference of four non-coplanar laser beams in a film photoresist. Unfortunately, the structures created by this method may have wetting and spreading problems when used in biomedical studies. Dip-pen nanolithography^7^, however, is a direct writing method, which uses an ‘ink’-coated AFM tip to pattern a great many soft materials on a variety of surfaces with a resolution of ≤50 nm. However, despite the high resolution, this process is confined to two-dimensional applications.

Dual-photon photochemical crosslinking is an emerging technology utilizing femtosecond lasers for rapid free-form writing^8^,^9^,^10^,^11^. It is mould-free and has several advantages for biomedical applications over more traditional methods, such as photolithography^12^ and micro-contact printing^13^, especially with regards to the spatial and temporal control that can be achieved^14^. Additionally, the processing conditions are free of any harsh organic solvent or cross-linker, which can deteriorate biocompatibility, and a resolution of ~100 nm can be accomplished due to the two-photon absorption capability. This method can therefore achieve the nano-sized features that are necessary to address the current tissue engineering challenges^3^. We recently described a multiphoton-based fabrication platform of user-defined protein microstructures and micropatterns with submicron features, controllable voxels, morphology, topology and porosity^15^, and demonstrated that it can be used as a simple “write-and-seed” miniaturized culture platform for cell niche studies. It has the major advantage of being a one-step simple and biocompatible system as it is mould-, coating- and label-free. As the microstructures and micropatterns are made of proteins, this fabrication process is therefore both biocompatible and cytocompatible, such that physiologically relevant protein niches can be engineered. The success of this technique has already been demonstrated as cells have been shown to form adhesions and secret extracellular matrix on protein micropillar arrays^15^. However, whether our multiphoton-based fabrication platform can be used to control the mechanical properties of such arrays has not been studied. This is especially important as the mechanical properties of the microenvironment have been shown to regulate cell fate^16^ and activities such as traction force^17^.

Here, we investigated whether the mechanical properties of engineered protein micropillar arrays (in this case, micropillars of bovine serum albumin, BSA were fabricated) can be precisely controlled by varying the fabrication and reagent parameters. We also determined if the different mechanical properties generated, are associated with the structural properties such as porosity and pore size. Finally, using micropillar arrays with a fixed modulus but different stiffness, we demonstrated the application of this platform in the measurement of single cell traction forces in multiple cell types.

## Results

### Fabrication parameters controlling the reduced elastic modulus of protein microstructures

**Figure 1:**
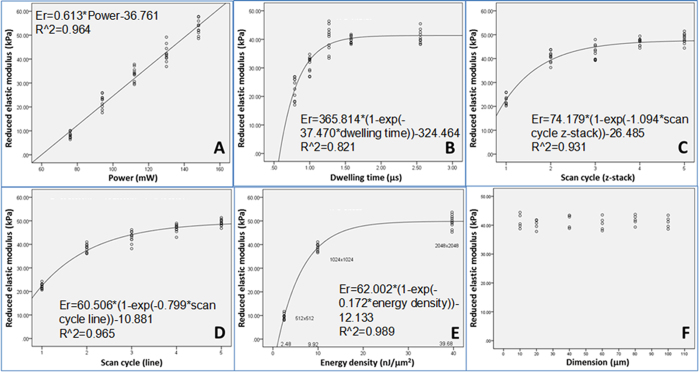
The effect of different fabrication parameters on the reduced elastic modulus of protein microstructures. The reduced elastic modulus was measured on square BSA matrices with a length of 40 μm and a height of 5 μm. Scatter plots showing the relationship between the reduced elastic modulus of the protein micropatterns and: (**A**) the scanning power (R^2^ = 0.9640, p < 0.001); (**B**) the scanning speed (R^2^ = 0.8212, p < 0.001), which is the scanning time on a single pixel; (**C**) the number of scanning cycles for the z-stack (R^2^ = 0.9305, p < 0.001); (**D**) the number of scanning cycles on a single line (R^2^ = 0.9650, p < 0.001); (**E**) the frame size (R^2^ = 0.9886, p < 0.001), which is proportional to the energy per pixel; and (**F**) the size of the protein microstructures (n = 10).

Figure 1 shows the effect of various fabrication parameters on the reduced elastic modulus of the BSA microstructures generated. As the scanning power increased from 75 mW to 147 mW, the reduced elastic modulus of the protein microstructure also increased from <10 kPa to >50 kPa (Fig. 1A). A strong positive linear relationship with a significant linear regression coefficient of 0.964 (p < 0.001) was detected between the scanning power and the reduced elastic modulus. Figure 1B–F shows that non-linear exponential recovery associations were found between the reduced elastic modulus and other fabrication parameters, including the dwelling time (Fig. 1B), scan cycle per z-stack (Fig. 1C), scan cycle per line (Fig. 1D), and the energy density (Fig. 1E). Specifically, when the dwelling time on a single pixel (or scanning speed) was increased to 1.5 μs, the reduced elastic modulus peaked at ~40 kPa and it was saturated thereafter (Fig. 1B). A statistically significant non-linear relationship between the dwelling time and the reduced elastic modulus was detected (exponential curve fitting, R^2^ = 0.8212, p < 0.001). The ‘scan cycle per z-stack’ refers to the number of cycles used to scan the entire z-stack and is proportional to the overall scanning duration. When the number of cycles increased to 5, the reduced elastic modulus of the BSA microstructures were saturated at ~45 kPa (Fig. 1C). A statistically significant non-linear relationship between the scan cycle per z-stack and the reduced elastic modulus was detected (exponential curve fitting, R^2^ = 0.9305, p < 0.001). Figure 1D shows the relationship between the scan cycle per line, which refers to the number of scans conducted on the same line (which is proportional to the duration stay on the same line) and the reduced elastic modulus. Similar to the scan cycle per z-stack, when the number of scans per line was increased to 5, the reduced elastic modulus also saturated at ~45 kPa kPa (Fig. 1D). A statistically significant non-linear relationship between the number of scans on the same line and the reduced elastic modulus was detected (exponential curve fitting, R^2^ = 0.9650, p < 0.001). Figure 1E shows the effect of energy density, which is controlled by the frame size (or pixel resolution) on the reduced elastic modulus. When the frame size was changed from 512*512 to 2048*2048, this resulted in a dramatic increase in the energy density per pixel from 2.48 nJ/μm^2^ to 39.68 nJ/μm^2^, which resulted in a saturation of the reduced elastic modulus of ~50 kPa (Fig. 1E). A statistically significant non-linear relationship between the frame size and the reduced elastic modulus was detected (exponential curve fitting, R^2^ = 0.9886, p < 0.001). Figure 1F shows the relationship between the size of the protein microstructures and the reduced elastic modulus. When the lateral dimension of microstructures was varied from 10 μm to 100 μm, there was no significant change in the reduced elastic modulus (one-way ANOVA, p = 0.84), suggesting that their size does not affect this intrinsic structural parameter.

### Reagent parameters controlling the reduced elastic modulus of protein microstructures

**Figure 2:**
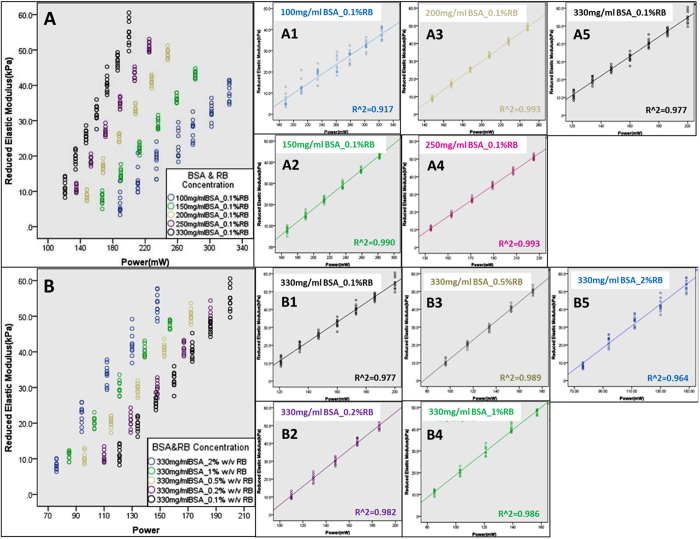
Effect of reagent concentration on the reduced elastic modulus of the protein micropatterns. (**A**) Scatter plot showing the relationship between the reduced elastic modulus of the BSA micropillars and the laser scanning power at different [BSA] and a constant [rose Bengal] ([RB]) of 0.1% w/v. (A1–A5) Linear regression plots to show the coefficients of determination (R^2^) at: (A1) 100 mg/ml, (A2) 150 mg/ml, (A3) 200 mg/ml, (A4) 250 mg/ml and (A5) 330 mg/ml BSA. (**B**) Scatter plot showing the relationship between the reduced elastic modulus of the BSA micropillars and the laser scanning power at different [RB] and a constant [BSA] of 330 mg/ml. (B1–B5) Linear regression plots to show the coefficients of determination (R^2^) at: (B1) 0.1%, (B2) 0.2%, (B3) 0.5%, (B4) 1.0%, and (B5) 2.0% (w/v) RB. (n = 10).

Figure 2 shows the dependence of the reduced elastic modulus of the protein microstructures on various reagent parameters. Figure 2A shows a scatter plot of the reduced elastic modulus against the scanning power at various [BSA] ranging from 100 mg/ml to 330 mg/ml. Simple linear regression analyses (Fig. 2A1–A5) showed that there was significant linear association between the reduced elastic moduli and the laser scanning power at all BSA concentrations because the coefficients of determination, R^2^, for simple linear regression analyses in all groups were >0.900 (p < 0.001). In addition, the slopes of the lines generated between the reduced elastic modulus and laser power (or the rate of change in the mechanical properties per unit laser power), increased in a linear manner with the increase in [BSA]. Furthermore, the range of laser scanning powers that could be used to fabricate micropillar arrays with a controllable reduced elastic modulus but without “burning” became narrower with the increase in [BSA]. For example, at a [BSA] of 100 mg/ml, the laser power working range for fabricating micropillars was 185–335 mW, but at a [BSA] of 330 mg/ml, the range was just 120–190 mW (Fig. 2A). Moreover, the threshold scanning power (i.e., the lowest power able to successfully fabricate protein micropillar arrays), was significantly lower at a high [BSA] such as 330 mg/ml (i.e., ~120 mW), than at a low [BSA] such as 100 mg/ml (i.e., ~190 mW). Figure 2B are scatter plots showing the relationship between the reduced elastic modulus and the laser scanning power at various concentrations of the photosensitizer, rose Bengal (RB), ranging from 0.1% to 2.0% (w/v) and a constant [BSA] of 330 mg/ml. Simple linear regression analyses showed that there was significant linear association between the reduced elastic moduli and the laser scanning power at all [RB] because the coefficients of determination (R^2^) for simple linear regression analyses in all groups were well above 0.900 (p < 0.001) (Fig. 2B1–B5). The slopes of the linearly associated curves between the elastic moduli and the laser power, or the rate of change in the mechanical properties per unit laser power, were constant as the [RB] was increased. This indicates that RB was not a limiting factor in the photochemical crosslinking process, which confirms previous reports that it is an efficient photosensitizer with high quantum yield^18^ even when used at low concentrations (i.e., 0.1% (w/v)). This suggests that the photoproducts responsible for the photochemical crosslinking produced after photon absorption, may already be in excess. The net range of the laser scanning power used to successfully fabricate micropillar arrays with controllable reduced elastic modulus but without “burning” was similar at all [RB]. For example, at an [RB] of 0.1%, the working range of the laser power required to fabricate micropillars was 120–200 mW (i.e., ~80 mW net range), and at an [RB] of 2%, it was 75–150 mW (i.e., ~75 mW net range) (Fig. 2B). It is obvious that the threshold scanning power, (i.e., the lowest power able to successfully fabricate protein micropillar arrays), was significantly lower at 2% RB (~75 mW) than at 0.1% RB (~120 mW).

### Scanning power and reagent concentrations are inversely associated with porosity and the pore size of the protein microstructures

**Figure 3:**
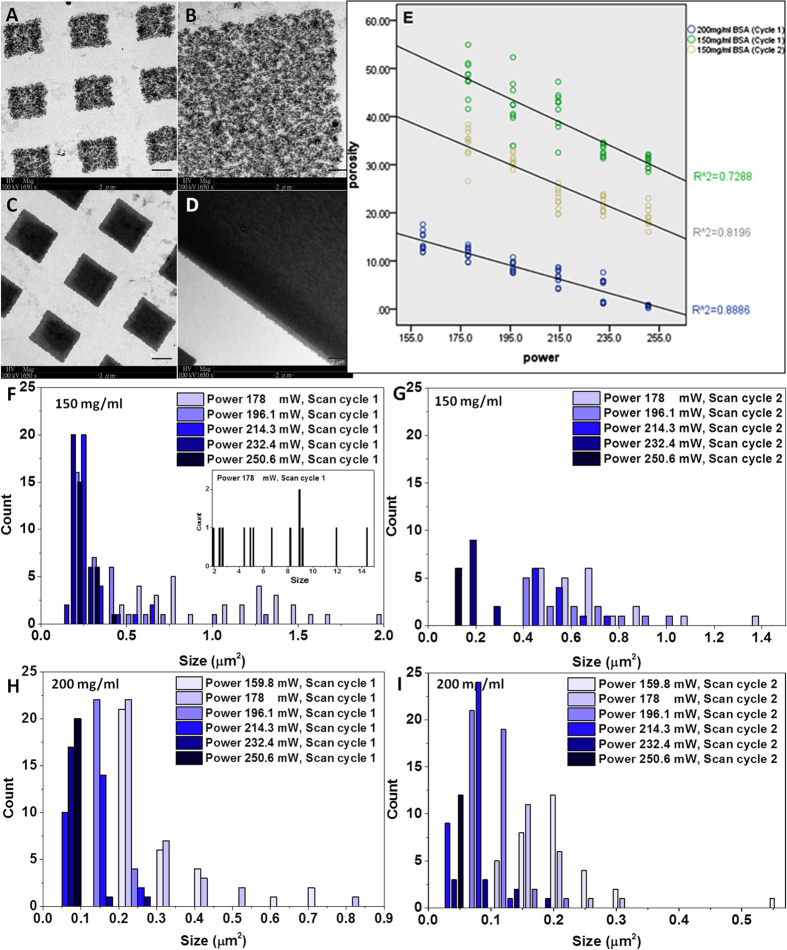
Porosity analysis of the protein micropatterns. (**A–D**): Transmission electron microscopy (TEM) images of (**A,C**) the protein micropillars and (**B,D**) the micromatrices at a [BSA] of (**A,B**) 100 mg/ml and (**C,D**) 300 mg/ml. Scale bars, 2 μm. (**E**) Scatter plot showing the effect of increasing the laser power (from 150 mW to 250 mW) on the porosity of the micromatrices at [BSA] of 150 mg/ml or 200 mg/ml, and 1 or 2 scanning cycles (n = 10). (**F–I**) Bar charts showing the size distribution of the top 0.1% largest pores of the protein micromatrices at different laser scanning powers ranging from 178 mW to 250.6 mW at [BSA] of (**F,G**) 150 mg/ml or (**H,I**) 200 mg/ml and (**F,H**) 1 or (**G,I**) 2 scanning cycles (n = 9–46).

In order to understand whether the multiphoton photochemical crosslinking-induced changes in mechanical properties of the protein microstructures are associated with a similar trend in the structural properties, a porosity analysis was conducted using TEM. Figure 3A–D show representative TEM images of the protein microstructures at different [BSA] at both low and high magnification. At a [BSA] of 100 mg/ml (Fig. 3A,B), the porosity of the protein structures was higher than that at a [BSA] of 300 mg/ml (Fig. 3C,D). Figure 3E shows the change in porosity of the BSA microstructures as the laser scanning power was increased from ~150 to ~250 mW, at 150 mg/ml or 200 mg/ml BSA and with scanning cycles of 1 or 2. Negative linear associations between the scanning power and the porosity were found at the different [BSA] and the different number of scanning cycles. The data show that as the scanning power was increased, then the porosity of the protein microstructures decreased. Increasing the number of scanning cycles from 1 (R^2^ = 0.7288, p < 0.001) to 2 (R^2^ = 0.8196, p < 0.001) at a fixed [BSA] of 150 mg/ml reduced the range of porosities throughout the entire spectrum of scanning power used. For example, with 1 scanning cycle, the porosity decreased from ~50% to ~30% (i.e., a 20% range) but at 2 scanning cycles the porosity decreased from just ~35% to ~20% (i.e., a 15% range) over the same scanning power spectrum used. The range of porosities was also significantly reduced throughout the entire spectrum of scanning power used when the [BSA] was increased from 150 mg/ml to 200 mg/ml at a constant scanning cycle of 1. Thus, at a [BSA] of 150 mg/ml, the porosity decreased from ~50% to ~30%, whereas at 200 mg/ml, it decreased from ~15% to ~0%. In addition to the porosity percentage, the pore size distribution in the protein microstructures was also analyzed (Fig. 3F–I). Specifically, the size of the top 0.1% largest pores of each sample was measured. The general trend for all the groups was that the pore size decreased as the laser power increased (Fig. 3F–I). This is left-shift in the pore size distribution at the higher laser powers, is shown by the darker blue bars, which are located toward the left side of the graph. When the [BSA] was maintained at 150 mg/ml and a scanning cycle of 1 (Fig. 3F) or 2 (Fig. 3G) was used, the pores ranging from ~0.2–2.0 μm and from ~0.2–1.4 μm, respectively, thus a left-shift in the pore size was observed with a scanning cycle of 2. A similar trend was shown when the [BSA] was fixed at 200 mg/ml (Fig. 3H,I). Increasing the scanning cycle from 1 (Fig. 3H) to 2 (Fig. 3I) resulted in a left-shift of the range of pore sizes from 0.05–0.8 μm (Fig. 3H) to 0.03–0.5 μm (Fig. 3I), respectively. Keeping the scanning cycle number at 1, and increasing the [BSA] from 150 mg/ml to 200 mg/ml also resulted in a left-shift in the pore size distribution from a range of 0.2–2.0 μm (Fig. 3F) to a range of 0.05–0.8 μm (Fig. 3H), respectively. A similar trend was also demonstrated when the scanning cycle was fixed at 2 and the [BSA] was increased from 150 mg/ml to 200 mg/ml (Fig. 3G,I), such that there was a left shift of the pore size distribution from 0.2–1.4 μm to 0.03–0.5 μm, respectively.

### Single cell traction force measurement

**Figure 4:**
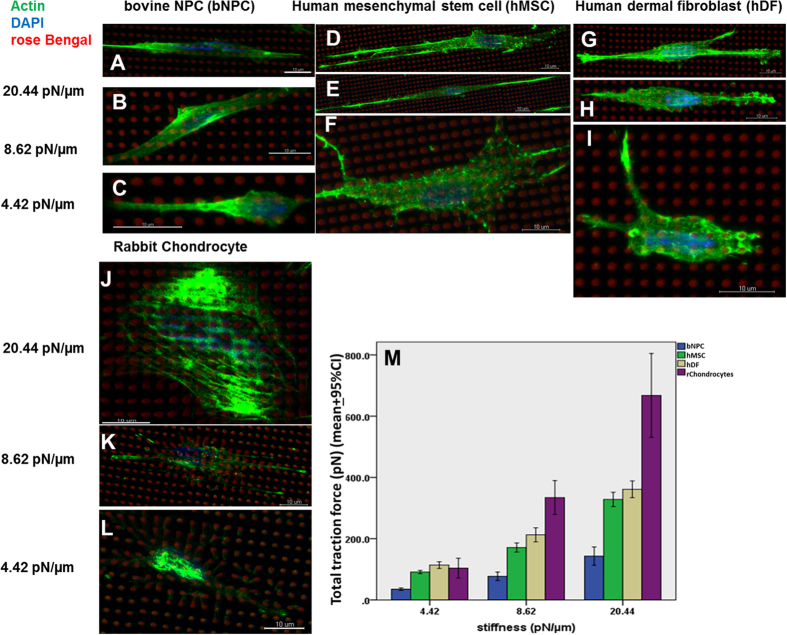
Single cell traction force measurement in multiple cell types using the protein micropillar arrays. (**A–I**) Fluorescence/Immunofluorescence staining of (**A–C**) bovine nucleus pulposus cells (bNPCs), (**D–F**) human mesenchymal stem cells (hMSCs), (**G–I**) human dermal fibroblasts (hDFs) and (**J–L**) rabbit chondrocyte, which were cultured on the protein micropillar arrays. Green: Immunofluorescence staining of beta-actin; Blue: DAPI showing the nuclei; Red: Rose Bengal showing the cross-sections of the protein micropillars. The micropillars all have a diameter of 1 μm and a reduced elastic modulus of 30 kPa, but are of different heights (i.e., 6 μm, 8 μm and 10 μm), which are equivalent to a stiffness of (**A,D,G,J**) 20.44 nN/μm, (**B,E,H,K**) 8.62 nN/μm, and (**C,F,I,L**) 4.42 nN/μm, respectively. Scale bars, 10 μm. (**M**) Bar chart showing the single cell total traction force of different types of cells cultured on protein micropillar arrays of different stiffness: bovine nucleus pulposus cells (bNPCs, P2 ~ P4); human mesenchymal stem cells (hMSCs, P3 ~ P5); human dermal fibroblasts (hDF, P5 ~ P15) and rabbit chondrocytes (rChondrocytes, P2 ~ P4) cultured for 3 days on protein micropillars (n = 10).

It has previously been reported that by keeping the reduced elastic modulus and the width of protein micropillars constant, their stiffness can be controlled by adjusting their height^19^. We showed that by keeping the reduced elastic modulus and the width at 30 kPa and 1 μm, respectively, protein micropillars with heights of 6 μm, 8 μm and 10 μm had a stiffness equivalent to 20.44 pN/μm, 8.62 pN/μm and 4.42 pN/μm, respectively. Figure 4A–L shows representative images of actin fluorescence staining revealing the morphology of bovine nucleus pulposus cells (bNPCs) (Fig. 4A–C), human mesenchymal stem cell (hMSC) (Fig. 4D–F), human dermal fibroblasts (hDFs) (Fig. 4G–I) and rabbit chondrocyte (Fig. 4J–L) on micropillars of different heights (and thus stiffness). All cell types exhibited a larger spread and elongated morphology, and the presence of stress fibers when cultured on micropillar arrays with high stiffness (i.e., 20.44 pN/μm; Fig. 4A,D,G,J). When cultured on protein micropillars with lower stiffness (i.e., 8.62 pN/μm), the cells showed reduced spread without stress fibers (Fig. 4B,E,H,K). On micropillars with very low stiffness (i.e., 4.42 pN/μm; Fig. 4C,F,I,L), all cell types exhibited a dendritic morphology with smaller spread. Figure 4M shows the traction forces of four different cell types: bNPCs, hDFs, human mesenchymal stem cells (hMSCs) and rabbit chondrocytes (rchondrocytes), which were measured when the cells were cultured on protein micropillar arrays of different stiffness. Specifically, as the stiffness of the micropillars increased, then the traction force of each cell type increased in a linear manner. Simple linear regression analyses showed that the relationship between the stiffness of the protein micropillars and the traction force was significant in all the cell types tested, with coefficients of determination as follows: bbNPCs (R^2^ = 0.738, p < 0.001), hMSCs (R^2^ = 0.947, p < 0.001), hDFs (R^2^ = 0.905, p < 0.001) and rchondrocytes (R^2^ = 0.779, p < 0.001). Looking at the magnitudes of the traction force generated by the different cell types, the rchondrocytes were the most sensitive to micropillar stiffness in that they generated a low traction force of around 100 pN on micropillars with a stiffness of 4.42 pN/μm, but a very large traction force of >600 pN on micropillars with stiffness of 20.44 pN/μm. On the other hand, hDFs and hMSCs generated a similar traction force of ~100 pN on the softest micropillars and <400 pN on the stiffest micropillars, whereas bNPCs generated the lowest traction forces of all the cells with value of ~50 pN and ~160 pN on the softest and stiffest micropillars, respectively. Two-way ANOVA showed that both the cell type (p < 0.001) and the stiffness of the micropillars (p < 0.001) significantly affected the traction force. Bonferroni’s posthoc tests showed that apart from the comparison between the hDFs and hMSCs (p = 0.345), all other pairs of comparison were statistically significant (p < 0.001).

## Discussion

The reduced elastic modulus was well controlled by varying a series of fabrication and reagent parameters. The strong linear relationship between the laser scanning power and the reduced elastic modulus demonstrated that this is one of most effective parameters for modulating the mechanical properties of protein microstructures during this multiphoton-based microfabrication process. The protein micropillars showed inverse (negative) linear association with porosity, suggesting that the control of reduced elastic modulus is likely to be mediated by the change in porosity of the microstructures. Other process controlling parameters such as scan cycle per z-stack or per line, dwelling time and energy density showed non-linear association with the reduced elastic modulus. Increasing the number of scanning cycles per z-stack initially increased the reduced elastic modulus prior to saturation. This saturation may be caused by the limited diffusion of free crosslinkable BSA molecules into the same spot after several cycles of crosslinking, which might result from the increased diffusion barrier owing to the decreased porosity of the protein microstructures fabricated. Specifically, increasing the scanning cycle from 1 to 2 dramatically decreased the porosity to <10%, prohibiting further crosslinking at the same site. The ‘scan cycle per line’ parameter was similar to the “scan cycle per z-stack” parameter and therefore showed a similar non-linear and saturation trend. When changing the frame size from 512*512 to 2048*2048, the energy density (energy per unit area) was dramatically increased to 39.68 nJ/μm^2^, which is approximately one-fold greater than the energy density following five cycles of scanning. Increasing the energy density directly resulted in an increase in both the number of photons reaching the same pixel area and the magnitude of crosslinking, and hence the reduced elastic moduli. Increasing the [BSA] was another effective way to control the reduced elastic modulus of the protein microstructures. However, controlling the reduced elastic modulus by altering different effective parameters simultaneously, (e.g., laser power and [BSA]) significantly changed the porosity of the microstructures. Since cell spreading and migration in 3D collagen matrices are reported to be more dependent on the porosity and less dependent on the stiffness of the substrate^20^, further studies are required to decouple the reduced elastic modulus from the porosity before their respective influence on cell behavior can be investigated. Additionally, at low [BSA], the maximal reduced elastic modulus was reduced; and this decrease could not be compensated by increasing the scanning power, which indicates that the availability of free crosslinkable BSA molecules were a limiting factor. When varying the [RB] from 0.1% to 2.0% (w/v), the range of reduced elastic modulus was almost constant. This might be because RB is known to be an efficient photosensitizer with high quantum yield that works well at low concentrations^18^ and the indirect mechanism involving singlet oxygen may be more dominant than the direct mechanism consuming the RB photosensitizer^18^.

One useful application of the protein micropillar arrays fabricated with this multiphoton platform is to measure the traction force generated by a single cell. In this study, the traction forces of different types of cells were measured on BSA micropillars with different stiffness. The phenotype of NPCs is known to be highly sensitive to the mechanical properties of the substrate^21^, for example substrates comprised of soft extracellular matrix have been shown to promote changes in NPC morphology, organization and phenotype^21^. One of the advantages of the current platform is that it can fabricate protein micropillar arrays with very low stiffness, providing an extremely soft environment for mechanical niche studies of cells such as NPCs. The traction force of both fibroblasts^17^ and human mesenchymal stem cells^22^ has been well studied by culturing them on polydimethylsiloxane (PDMS) microposts. However, cells cannot usually bind to PDMS directly, and (as cell adhesion is a pre-requisite to traction force generation) fibronectin is therefore also required. Because of the initial enhancement of adhesion through fibronectin-integrin binding, the influence of stiffness on traction force may thus be magnified (Supplementary Figure 1). Another advantage of the current platform is that cells can attach to the BSA microstructures directly, where they exhibit a range of physiological functions such as integrin-based adhesion and deposition of extracellular matrix, without the need to use exogenous extracellular matrix^9^; the cell culture studies are thus simplified considerably. Another advantage of this system is that the photosensitizer, RB, is a fluorescent molecule. It therefore serves as an intrinsic marker of the protein microstructures being fabricated, and thus the need for additional labeling steps (which are necessary in the PDMS micropost platform), is effectively eliminated in the current platform. Traction force is a measurement of the ability of a cell to contract the substrate, and it can therefore be used as a functional evaluation of cellular machineries such as cell adhesion components and the cytoskeleton^23^. Moreover, cells at different levels of maturity, or developmental or differentiation stages are able to interact with the environment in different ways, and hence they might generate distinct traction forces. Examples include immature cardiomyocytes differentiating into mature cardiomyocytes^24^; notochord cells, which are immature progenitor cells present in developing embryos that develop into NPCs in the mature intervertebral disc^21^, and mesenchymal stem cells, which become committed to the chondrogenic lineage by differentiating into osteochondral progenitors, prechondrocytes, mature chondrocytes and hypertrophic chondrocytes^25^. As a result, this platform can serve as a functional evaluation for cells of different maturity during development and differentiation.

Our previous work described the multiphoton photochemical crosslinking bio-fabrication technology in detail, and demonstrated its controllability over morphology, topology and porosity of protein microstructures^15^. In this current study, we have further developed the platform so that now we have the capability to precisely control the mechanical properties of engineered microtissues, including the intrinsic material properties such as the reduced elastic modulus, and the environmental substrate compliance such as stiffness. We are now able to create protein micropatterns with defined or even a gradient of mechanical properties for studying cellular activities and cellular fate processes including adhesion, migration, proliferation and differentiation. Ongoing in-house studies are now establishing the ability of our system to spatially control the type of matrix proteins in the protein micropatterns, and investigating the phenotype maintenance of different types of cells on protein micropillar arrays by utilizing different combinations of topological and mechanical properties with different extracellular matrix proteins. The current platform represents a truly 3D microprinting, highly versatile and precisely controllable system for cell niche studies.

## Methods

### Two-photon photochemical crosslinking system

A two-photon confocal laser scanning microscopy system (Zeiss 710, Carl-Zeiss, GmbH, Jena), equipped with a mode-locked Ti:Sapphire femtosecond near infrared (NIR) laser (Coherent, Inc., Santa Clara, California, USA) was used to fabricate the protein microstructures. The laser was tuned to 800 nm and a 40x/1.3 N.A. oil-immersion objective lens was used to aid the multiphoton fabrication process. The laser power was measured using a power meter (Coherent, Inc.) before each fabrication.

### Sample loading and autofocusing

Glass-bottomed 35 mm confocal culture dishes (P35G-1.5-10-C, MatTek Corp., Ashland, MA, USA) were used as the substrate for fabrication. Each confocal dish was sterilized under UV light for 15 min before use. Fifty μl aliquots of BSA (Sigma-Aldrich Corp., St Louis, MO, USA) and the photosensitizer, rose Bengal (RB; Sigma-Aldrich) were mixed at pre-determined ratios and then loaded into the center of the confocal culture dish. The dish was mounted onto the stage of the inverted Zeiss 710 microscope, after which the autofocus function in the Z-710 reflection mode was used in conjunction with an Argon laser at 488 nm excitation to find the liquid-solid interface between the protein solution and the glass bottom of the culture dish. The interface was marked as the zero position in the z-direction.

### Preparation of the reagents

Stock solutions of RB (at 2% w/v in 1xPBS) and BSA (at 367 mg/ml in 1xPBS) were freshly prepared prior to each experiment. Solutions of BSA and RB at different working concentrations were prepared as follows: BSA was prepared at 100 mg/ml, 150 mg/ml, 200 mg/ml, 250 mg/ml and 330 mg/ml, whereas RB was prepared at 0.1%, 0.2%, 0.5%, 1% and 2% (w/v).

### Fabrication parameters

BSA and RB were mixed at the desired concentrations and then loaded onto the sample stage for the fabrication of the microstructures. Square protein matrices of different lengths ranging from 10 μm to 100 μm were fabricated to study whether the mechanical properties of the protein matrices are dependent on matrix dimension. Fabrication parameters, such as scanning power, scanning speed or dwelling time (the scanning time in a single pixel), the number of scanning cycles in a z-stack (the scanning time of the entire z-stack fabrication), the number of scanning cycles in a single line (the scanning time of a single line), and the frame size (which is proportional to the energy density per pixel) were varied to study their association with the reduced elastic moduli of the protein micropillars. Reagent parameters, such as the [BSA] and [RB] were also varied to study their influence on the reduced elastic modulus. The other fabrication parameters were kept constant unless otherwise specified. These are as follows: z-stack interval (i.e., the distance between two adjacent layers) = 0.5 μm, scanning speed = 1.27 μs, cycle = 1, number = 1, scanning zoom = 2.1 (i.e., 101 μm*101 μm), frame size = 512*512 (i.e., pixel size 0.2 μm*0.2 μm), [BSA] = 330 mg/ml, and [RB] = 0.1% w/v.

### Fabrication of protein micropillar arrays for cellular studies

Protein micropillars that were cylindrical in shape were fabricated by using the region of interest (ROI) function of the microscope software. The micropillars had a diameter of 1 μm and there was a gap of 2 μm between adjacent micropillars. The scanning start position was set at 1 μm below the interface to make sure that micropillars were firmly attached to the bottom of the dish, whereas the scanning end position was set at 6 μm, 8 μm and 10 μm above the interface. The fabrication parameters were set as follows: scanning power = 75 mW, scanning speed = 1.27 μs/pixel, number = 1, cycle = 1, z-stack interval = 0.5 μm, [BSA] = 330 mg/ml and [RB] = 0.1% w/v.

### Measurement of elastic modulus

**Figure 5:**
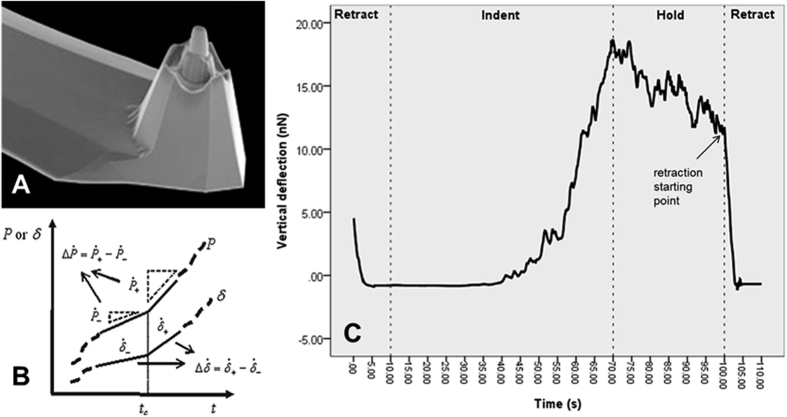
Atomic force microscopy (AFM) nano-indentation. (**A**) The AFM indenter tip (PL2-CONT, Nanosensor) with a plateau on the top; (**B,C**) Line graphs to show (**B**) the rate-jump of force (P) and displacement (δ) at time t_τ_; and (**C**) the vertical deflection (nN) over time (s).

The reduced elastic modulus of the protein micropillars was measured using an atomic force microscope (AFM)-based rate-jump method^26^ as shown in Fig. 5. The AFM probes (PL2-CONT, Nanosensor) used were chemically inert, of high sensitivity, and had a plateau with a typical diameter of ~1.8 μm (Fig. 5A). The overall height of the tip was 10–15 μm and the height of the plateau rod was >2 μm. The force constant of the cantilever was 0.02 ~ 0.77 N/m with a typical value 0.06 N/m in aqueous medium. Most biological and soft materials exhibit nonlinear viscoelastic behaviors under load, and the constitutive law of such materials consist of dashpot as well as elastic-spring elements, which correspond to the viscous and elastic components of the deformation, respectively. In the rate-jump method, the load and displacement are made to undergo step-wise changes in their rates, and it has been shown that the resultant jumps in the rate of the stress, strain and/or displacement satisfy a constitutive law in which the elastic-springs are identical to the original viscoelastic law of the material, but with the dashpot elements removed^26^. Hence, by replacing the stress, strain and/or displacement with their rate-jumps, the original nonlinear viscoelastic problem can be reduced to a relatively simple linear elastic problem, which can be solved analytically or numerically. In the AFM-indentation configuration, after replacing the displacement and the force with their jumps of loading rate, the relationship between the rate-jumps of displacement and loading force was expressed by^26^


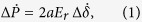


where *P* is force, *a* is the radius of the probe plateau, *δ* is the tip displacement, and *E*_*r*_ is the reduced elastic modulus of indentation (Fig. 5B). Since the elastic modulus of the AFM tip (in the hundreds of GPa range) is much larger than that of the BSA matrices (in tens of kPa range), *E*_*r*_ is given to a high enough accuracy as:


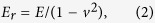


in which *v* and *E* represent the Poisson ratio and Young modulus of the BSA sample, respectively.

Thus, the reduced modulus *E*_*r*_ was first calculated from Eq. (1) from the rate-jump responses 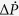 and 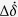 in the indentation tests, and then the Young modulus *E* of the sample was calculated from Eq. (2). In this work, the Poisson ratio ν of the BSA matrices was assumed to be 0.5.

The procedures used to measure the reduced elastic modulus are shown in Fig. 5C. Using a small reference force of ~4 nN, the AFM probe was moved to touch the surface of a microstructure. When the surface was slightly indented, time zero was set. In order to record the force and deformation data from the beginning of indentation, the probe was retracted by 1 μm from the surface at a speed of 0.1 μm/s and then moved to indent the sample surface at a speed of 0.02 μm/s for 1.2 μm. After indentation of 0.2 μm, the probe was held at constant height for 30 s to allow the indentation force to partially relax. The probe was then retracted by 1 μm again from the reference position at a loading speed of 0.1 μm/s. The starting point of the second retraction was used to derive the rate-jump of loading force and deformation. The reduced elastic modulus was calculated using the formula (2) above. In order to determine if the in-plane dimension might affect the reduced elastic modulus of the protein microstructures being fabricated, measurements were made on square BSA microstructures with different dimensions ranging from 10 μm to 100 μm at the sides and at a fixed height of 5 μm.

### Estimation of the stiffness of the protein micropillars

The bending stiffness of the protein micropillars, determined by measuring the horizontal traction force at their top, can be controlled by varying their height. The bending stiffness of the protein microstructures was calculated using the formula^19^:


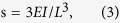


where *E* is the reduced elastic modulus measured at given fabrication and reagent parameters, *I* is the moment of inertial and equal to 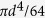 here and *L* is the height of the micropillars. Therefore, the stiffness for micropillars with 6, 8 and 10 μm heights was 20.44 pN/μm, 8.62 pN/μm and 4.42 pN/μm, respectively.

### Measurement of the porosity and pore size of the BSA microstructures

**Figure 6:**
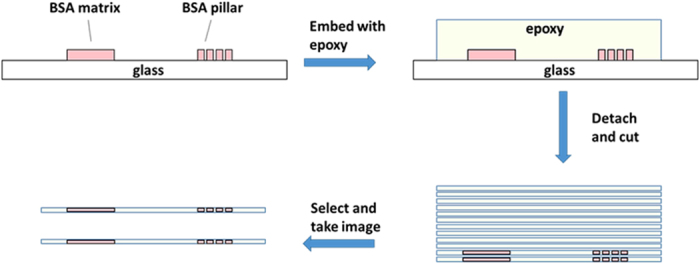
Schematic diagram illustrating the preparation of the BSA matrix and pillars for the porosity measurements.

Transmission Electron Microscopy (TEM) was used to measure the porosity of the BSA microstructures that had been fabricated with laser power ranging from 159.8 mW to 250.6 mW. The microstructures were then fixed with 2.5% glutaraldehyde, and then dehydrated through a gradient (i.e., 25%–50%–70%–80%–90%–100%) of increasing concentrations of ethanol, after which they were embedded in Epon 812 epoxy (SPI-CHEM, West Chester, PA) and then cut into slices of ~80–100 nm thickness (Fig. 6). Images were acquired with a Philips CM100 TEM (Eindhoven, Holland), and Image J (NIH, USA) was used to analyze the porosity and pore size. Ten regions of each sample were randomly chosen.for ‘threshold and particle analysis’ during porosity analysis. Measurements were made to determine the percentage of pores in relation to the total area analyzed, and the pore size, which was the area of the pores among the microstructures.

### Cell culture and reagents

Human dermal fibroblasts (hDFs) from neotissues were obtained from commercial source (CC-2509 NHDF-Neo, Lonza, Basel, Switzerland). Human MSCs (hMSCs) were obtained from the Texas A&M Health Science Center College of Medicine Institute for Regenerative Medicine (Scott & White Hospital, Bryan, TX, USA). Different types of cells including hDFs at P5 ~ P15, hMSCs at P3 ~ P5, and bovine NPCs (bNPCs) at P2 ~ P4 were cultured in growth medium containing low-glucose Dulbecco’s modified Eagle’s medium (DMEM, Invitrogen, Grand Island, NY), 10% fetal bovine serum (FBS, Invitrogen), and 100 μg/ml penicillin and 100 μg/ml streptomycin (pen/strep) (Invitrogen). Rabbit chondrocytes (rChondrocytes) at P2 ~ P4 were cultured in growth medium containing DMEM-HG (1.85 g/L NaHCO_3_), 4.766 g/L HEPES, 10% FBS, 0.292 g/L L-glutamine, and 1% pen/strep. Cells were seeded on BSA micropillars with a density of 1E5/ml, and cultured for 3 days prior to the traction force measurement and immunofluorescence staining for beta-actin. No extracellular matrix proteins were coated on the BSA micropillars.

### Measurement of traction force

Traction force was calculated using the following formula^19^:


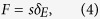


where *F* is traction force, *s* is bending stiffness defined in Eq. (3), and 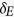 is the elastic part of the deflection of the micropillars. As these photochemical crosslinked BSA micropillars are viscoelastic materials, it was assumed that the Maxwell model can describe their deformation^27^. Therefore, the total stress 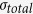 and total strain 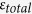 are given as


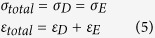


where ‘*D*’ and ‘*E*’ are dashpot and elastic spring, respectively.

**Figure 7:**
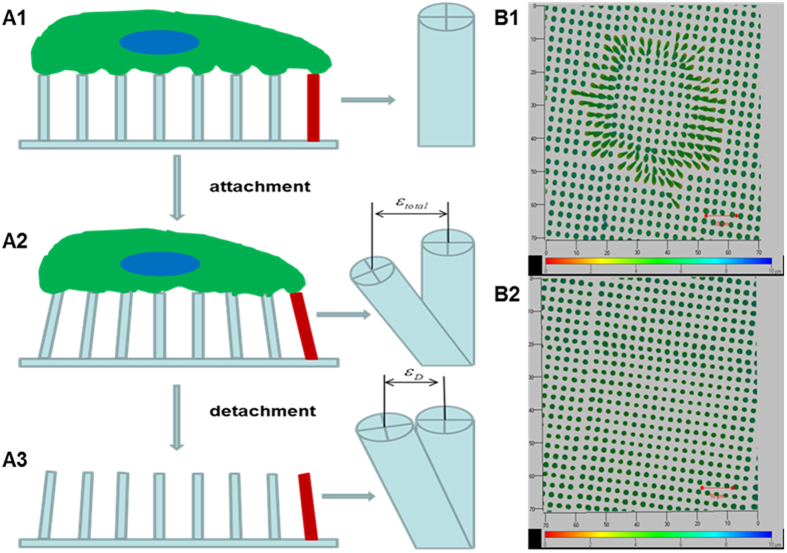
Measurement of the deflection of protein micropillars. (**A1**) The micropillars do not deflect just before attachement. (**A2**) The total strain (ε_total_) was measured by examining rose Bengal fluorescence of the micropillars after cell attachment. (**A3**) The viscous part of the total strain (ε_D_) was measured after cell detachment by trypsinization for 15 min at room temperature. The elastic part of the total strain (ε_E_) was calculated as ε_total_ minus ε_D_. (**B1**,**B2**) The bending of the micropillars with the color bar indicates the depth of the image (**B1**) before and (**B2**) after cell detachment. Scale bars, 10 μm.

Figure 7 shows the procedures used to measure the elastic part of deflection. The total strain 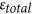 was measured by tracing the fluorescence of RB as the deflection of the micropillars before cell detachment (Fig. 7A2). Incubation with 0.25% trypsin for 15 min at room temperature was used to remove the cells from micropillars. The viscous strain component, 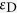, which is also the plastic component of the deformation, was measured by tracing the fluorescence of RB as the residual deflection of the micropillars after cell detachment (Fig. 7A3). Therefore, the elastic strain component, 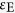, which is the recovery distance after cell detachment was calculated as 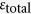 minus 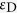. The bending of micropillars before and after cell detachment are shown in Fig. 7B1, B2, respectively. The color scale indicates the depth of imaging that was used to precisely find the top and the bottom of the micropillars. Unlike PDMS, protein microstructures are viscoelastic such that trypsinization is necessary to eliminate the viscous element of the micropillars. The influence of the trypsinization process (if any) on the reduced elastic modulus was therefore also evaluated by measuring this parameter after treatment with 0.25% trypsin at room temperature for 15 min and no significant change in the elastic modulus was found (Supplementary Figure 2).

### Immunofluorescence staining

For F-actin staining, samples were fixed with 4% paraformaldehyde for 10 min at room temperature and then washed with 1xPBS for 3 × 5 min. Cells were then permeabilized with 0.5% Tween 20 for 10 min, after which the samples were washed again with 1xPBS for 3 × 5 min. Samples were blocked in 5% BSA in PBS for 30 min and then incubated with a primary monoclonal anti-β-actin antibody (1:100, A2228, Sigma-Aldrich) at 4 °C overnight. They were then washed 3x with 1xPBS, after which they were incubated with secondary antibody (1:400, Invitrogen) for one hour. Samples were then washed three times with 1xPBS and mounted in Fluro-gel II (EMS) containing DAPI. Z-stack images were taken at 0.5 μm intervals using the Zeiss LSM710 confocal system with a 63x/1.4 N.A. objective lens (Plan-Apochromat) and analyzed with the 3D image reconstruction software Imaris (Bitplane, Zurich, Switzerland).

### Data analysis and statistics

Quantitative information regarding the dimensions of the protein microstructures, as well as the reduced elastic moduli and traction forces are presented as mean ± SEM. The normality assumption was verified with the Kolmogorov-Smirnov test and the equal variance assumption was verified by Levene’s test to justify the use of parametric tests. Simple linear regression analysis was conducted to determine the association between scanning power and the reduced elastic moduli, between the reagent concentration parameters and the reduced elastic moduli, between the porosity and different fabrication and reagent parameters, and between the cellular traction force and the stiffness of the protein micropillars. Non-linear curve fitting was conducted to determine the association between the reduced elastic moduli and fabrication parameters other than scanning power. The coefficient of determination (R^2^) was reported in all association studies. Two-way ANOVA (with the appropriate post-hoc tests) was used to reveal the differences in cell traction force when culturing cells on protein micropillar arrays of different stiffness and among different cell types. For data with equal variance assumed, Bonferroni’s test was used. For data without equal variances, Dunnett’s T3 test was used. SPSS 23.0 (Armonk, NY) and OriginPro 9 (OriginLab, Northampton, MA) were used to execute all analyses and the statistical significance was set at α = 0.05.

## Additional Information

**How to cite this article**: Tong, M. H. *et al.* Multiphoton photochemical crosslinking-based fabrication of protein micropatterns with controllable mechanical properties for single cell traction force measurements. *Sci. Rep.* **6**, 20063; doi: 10.1038/srep20063 (2016).

## Supplementary Material

Supplementary Information
