# Supplementary material for: Multiphoton photochemical crosslinking-based fabrication of protein micropatterns with controllable mechanical properties for single cell traction force measurements
Source: Sci Rep. 2016 Jan 28;6:20063. doi: 10.1038/srep20063 (PMC4730236; doi:10.1038/srep20063)
Supplement: Supplementary Information [file srep20063-s1.pdf]

Multiphoton photochemical crosslinking-based fabrication of protein micropatterns with controllable mechanical properties for single cell traction force measurements

Ming Hui Tong[1], Nan Huang[1], Wei Zhang[1], Zhuo Long Zhou[2], Alfonso Hing Wan Ngan[2], Yanan Du[3], Barbara Pui Chan[1,*]

[1]Tissue Engineering Laboratory, Department of Mechanical Engineering, The University of Hong Kong, Pokfulam Road, Hong Kong Special Administrative Region, China.

[2]Department of Mechanical Engineering, The University of Hong Kong, Pokfulam Road, Hong Kong Special Administrative Region, China.

[3]Department of Biomedical Engineering, School of Medicine, Tsinghua University, Beijing, 100084, China

*Corresponding author: bpchan@hku.hk

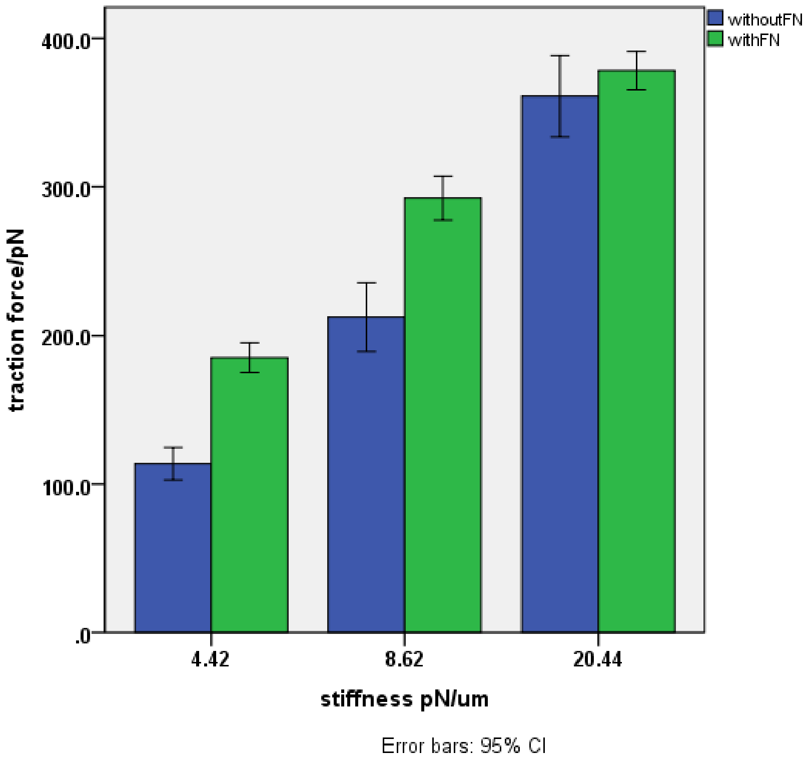

Supplementary Figure 1 – Traction force of human dermal fibroblast on micropillars with and without fibronectin coating.

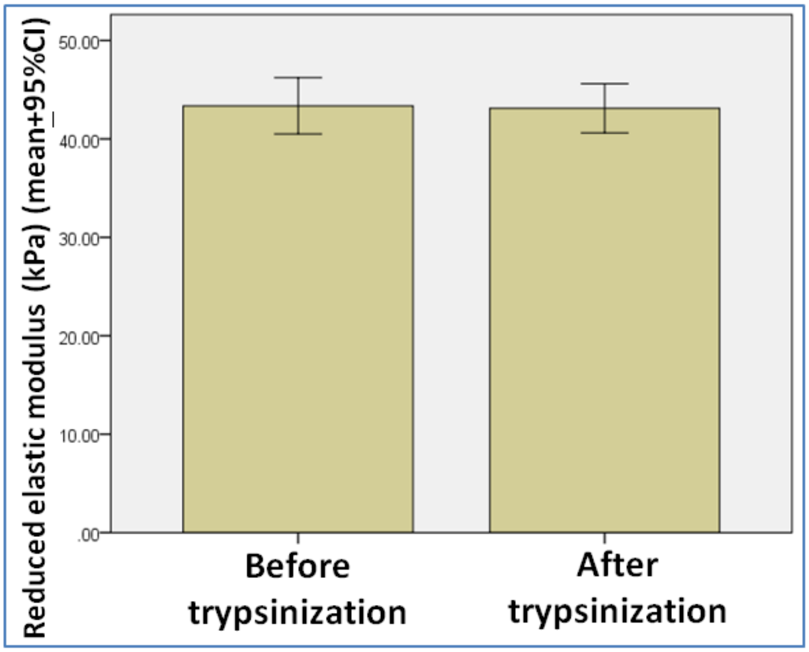

Supplementary Figure 2 – Measurement of the reduced elastic modulus before and after trypsinization.
